# Association between gene polymorphisms of voltage-dependent Ca^2+^ channels and hypertension in the Dai people of China: a case-control study

**DOI:** 10.1186/s12881-020-0982-9

**Published:** 2020-02-28

**Authors:** Lifan Huang, Yan Chu, Xiaoqin Huang, Shaohui Ma, Keqin Lin, Kai Huang, Hao Sun, Zhaoqing Yang

**Affiliations:** 1 Institute of Medical Biology Chinese Academy of Medical Sciences and Peking Union Medical College, Kunming, China; 2 Department of General Surgery of the 2nd People Hospital of Yunnan Province, Kunming, China

**Keywords:** Voltage-dependent calcium channels, Polymorphism, Hypertension, Dai people

## Abstract

**Background:**

Abnormal calcium homeostasis related to the development of hypertension. As the key regulator of intracellular calcium concentration, voltage-dependent calcium channels (VDCCs), the variations in these genes may have important effects on the development of hypertension. Here we evaluate VDCCs variability with respect to hypertension in the Dai ethnic group of China.

**Methods:**

A total of 1034 samples from Dai individuals were collected, of which 495 were used as cases, and 539 were used as controls. Blood pressure was measured using a standard mercury measurement method, three times with a rest for 5 min, and the average was used for analyses. Seventeen single nucleotide polymorphisms (SNPs) in the four protein-coding genes (*CACNA1A, CACNA1C, CACNA1S, CACNB2*) of VDCCs were identified by multiplex PCR-SNP typing technique. Chi-square tests and regression models were used to analyse the associations of SNPs with hypertension.

**Results:**

The results of chi-square tests showed that the allele frequencies of 5 SNPs were significantly different between the case and the control groups (*P* < 0.05), but the statistical significance was lost after Bonferroni’s correction. However, after adjusting for BMI, age, sex and other factors by logistic regression analyses, the results showed that 5 SNPs consistent with chi-square tests (rs2365293, rs17539088, rs16917217, rs61839222 and rs10425859) were still statistically positive.

**Conclusions:**

This finding suggested that the significant association of these SNPs with hypertension may be noteworthy in future studies.

## Background

Essential hypertension (hereinafter referred to as hypertension) is now a major risk factor for stroke, cardiovascular disease and end-stage renal disease [1]. Its prevalence presented a trend of rapid increase over the past decade, especially in less developed areas. According to data from the Non-Communicable Diseases Risk Factor Collaboration (NCD-RisC), the number of adults with hypertension reached 1.13 billion in 2015 [2]. In China, hypertension affects 28% of adults. A total of 82.9% of patients received treatment, but only 30% of the treated patients had their blood pressure effectively controlled [3]. Therefore, investigating the pathogenesis of hypertension for improving the prevention and treatment of hypertension and increasing the average life span of the population is important.

The relationship between hypertension and intracellular calcium concentration has been confirmed by a large number of experiments [4–7]. Therefore, changes in the function of calcium channel proteins, which have a regulatory effect on intracellular calcium concentration, may be closely related to the abnormal changes in blood pressure and the development of hypertension. Voltage-dependent calcium channels (VDCCs) [8] control the entry of calcium ions into excitable cells, causing depolarization and excitation-contraction coupling of vascular smooth muscle cells (VSMCs), which not only plays an important role in regulating the concentration of calcium but also determines the contractility in VSM cells [9, 10]. Although functional experiments have supported that VDCCs can regulate blood pressure in recent years [11, 12],whether variations in the genes of VDCCs affect the function of this ion channel and the development of hypertension remains unclear.

In this study, blood samples were extracted from subjects belonging to the Dai people, an ethnic group native to Yunnan province of China for centuries. As Dai people have special feelings for their land, one of their characteristics is to firmly hold on to their native land. Most of them live in the river valleys and flat areas surrounded by mountains. Therefore, the geographical barriers make it extremely difficult for them to communicate with groups beyond the boundary of Dai ethnic. Meanwhile, due to the closure of the society, the lack of understanding and even the estrangement with other ethnic groups, Dai people adopted the custom of intermarriage within their own group and rarely intermarried with other ethnic groups before the founding of New China. As a result, compared with other ethnic groups (such as Han), Dai people have relatively small genetic differences [13] and pathogenic spectrum among individuals.

## Methods

### Study subjects

The study was approved by the Ethics Committee of the Institute of Medical Biology Chinese Academy of Medical Sciences, and signed informed consent from all study subjects was obtained. The samples were collected from Dai ethnic groups living in Xishuangbanna and Dehong, Yunnan, China during 2015–2018. (Each member was traced backward to the same ethnic group for three generations and is not related to other members.) Since the prevalence of hypertension is related to age, the patients in this study were all over 40 years old (including 40 years old). The inclusion criteria of the hypertensive group were defined by the World Health Organization (1999) (systolic blood pressure (SBP) ≥140 and/or diastolic blood pressure (DBP) ≥90) or if they had previously diagnosed hypertension or were taking antihypertensive medication. Patients with renal and endocrine diseases that can cause secondary hypertension were excluded. The control group was SBP < 140 and DBP < 90, and there was no BP-lowering therapy. Blood pressure was measured using a standard mercury measurement method, three times with a rest for 5 min, and the average was used for analyses. Age, sex, body mass index (BMI), total triglyceride (TG), low density lipoprotein (LDL) and high density lipoprotein (HDL) were recorded for all subjects. A total of 1221 patients (over 40 years old) were recruited. Considering the possibility of measurement errors, we eliminated the samples with critical values (±5 mmHg). Finally, a total of 1034 patients were included, including 495 cases and 539 controls.

### Choosing SNPs and genotyping

**Fig. 1:**
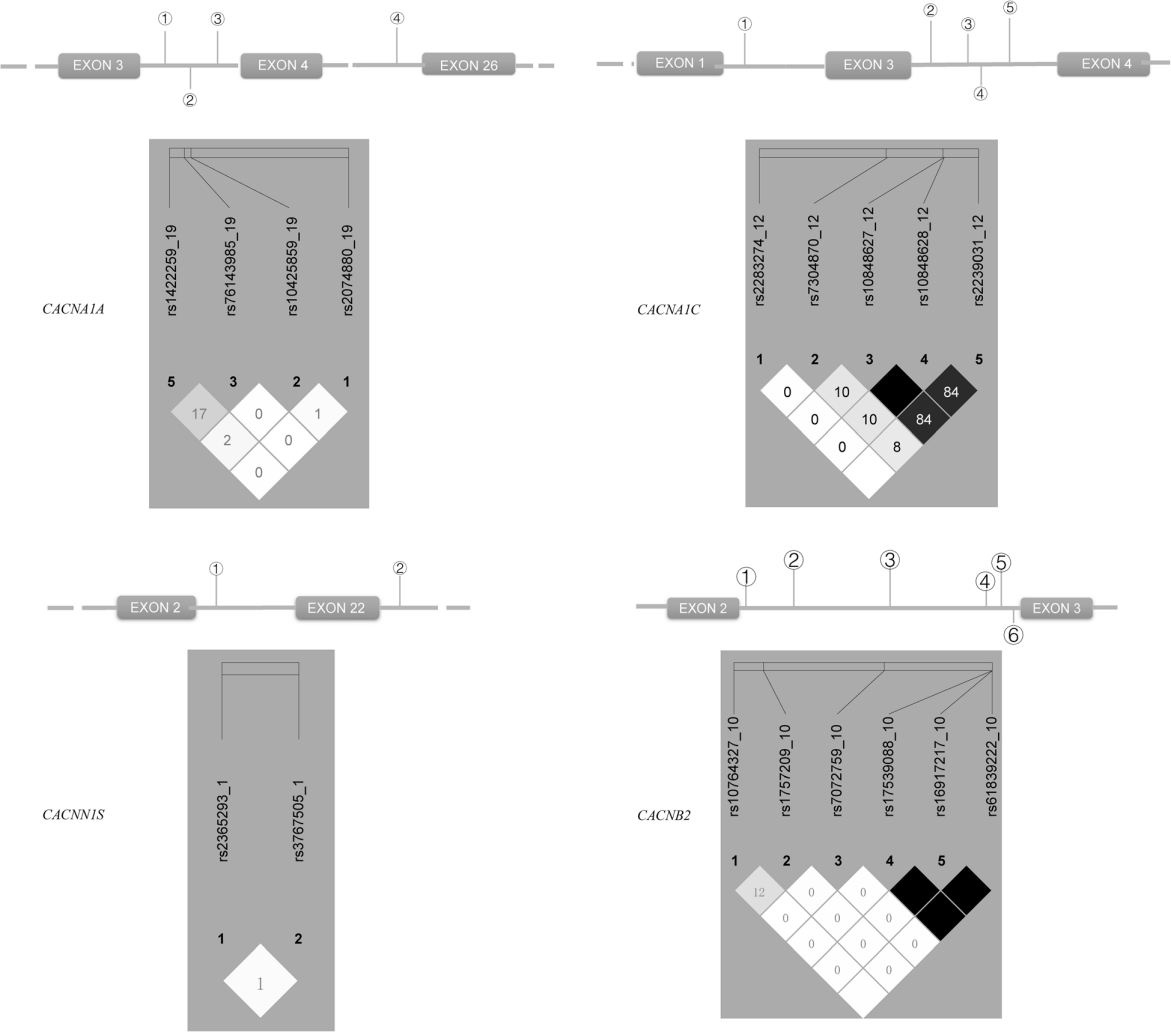
Locations of the 17 SNPs at the VDCC coding-gene locus and their haplotype configurations. The values shown in these haploblock figures are all r2 in Dai people

Although the association analyses in the Chinese Han population [14, 15] and other countries [16, 17] suggested that the *CACNA1A* (rs8182538), *CACNA1C* (rs758116) and the *CACNB2* (rs4373814, rs11014166, rs12258967) polymorphisms were associated with blood pressure and hypertension (no reports about *CACNA1S* polymorphisms), these SNPs were not consistent with our pre-experimental results (200 cases vs 200 controls, unpublished results). Therefore, based on the haplotype analysis results of the Beijing Han (Chinese Han in Beijing (CHB), HapMap) [18], we chose another 17 SNPs in the genes (*CACNA1A, CACNA1C, CACNA1S, CACNB2*) that encode the proteins of VDCCs to perform genotyping in the Dai population. The locations of these SNPs are shown in Fig. 1. There are 4 SNPs in *CACNA1A*, 5 SNPs in *CACNA1C*, 2 SNPs in *CACNA1S* and 6 SNPs in *CACNB2*. Linkage disequilibrium analysis of these SNPs was performed by Haploview software [19].

Peripheral blood (2 ml) of participants was extracted and placed into the heparin anticoagulation vacuum tube. DNA preparation: DNA extraction was performed using the AxyPrep Blood Genomic DNA Mini Prep Kit (Axygen, Hangzhou City, China) according to the manufacturer’s instructions. PCR primer design: Primer 3 online version 0.4.0 was used to design specific multiple primers, which were supplied by Shanghai Balig Biotechnology Co., Ltd. The primer sequences are shown in Supplementary Table 1. Multiplex PCR amplification was performed to obtain the PCR products (first round reaction mix for PCR contained 3.2 μl of ddH_2_O, 1 μl of 10X buffer, 2 μl of 50 nM primer, 0.8 μl of 2.5 mM dNTP, 0.1 μl of 5 U/ul Taq polymerase, 2 μl of DNA, 1 μl of 100 mM Mg^2+^). Reaction system conditions included pre-denaturation 95 °C for 15 min, denaturation at 94 °C for 30 s, annealing at 60 °C for 10 min, extension at 72 °C for 30 s for 4 cycles and an extension at 72 °C 30 s for 20 cycles. The second round PCR mix included 3.6 μl of ddH_2_O, 2 μl of 10X buffer, 3.6 μl of 2uM barcode, 0.8 μl of 2.5 mM dNTP, 0.1 μl of 5 U/ul Taq polymerase, 10 μl of DNA, and 1 μl of 100 mM Mg^2+^. Reaction conditions included pre-denaturation at 95 °C for 15 min, denaturation at 94 °C for 30 s, annealing at 60 °C for 4 min, extension at 72 °C for 30 s for 5 cycles and annealing at 65 °C for 1 min and extension at 72 °C for 30 s for 20 cycles). The Illumina HiSeq platform was used for sequencing and the sequencings were performed by Novogene Co., Ltd. (Beijing, China). The sequencing images were transformed into sequenced reads through base recognition analysis, and final identification of SNPs was conducted by Samtools 0.1.19 [20].

### Statistical analysis

After the genotypes of the SNPs were identified, we performed statistical analyses between the case and control groups. The quantitative data were described by mean ± standard deviation, and the comparison between the two groups was performed by independent-sample T test. Qualitative data and allele frequencies were compared using the chi-square test. The associations between SNPs and hypertension were further adjusted (adjusted age, sex, BMI, TG, LDL and HDL) by logistic regression analyses in three genetic models (dominant model (AA+AB vs BB, with minor A), recessive model (AA vs AB+BB), and additive model (AA vs AB vs BB))**.** The T test and chi-square test were performed using SPSS 25.0 software. Logistic regression analyses were performed by plink 1.9 software [21]. Relative risk was expressed by odds ratio (OR) and 95% CI. All statistical tests were statistically significant with two-tailed probability (*P* < 0.05).

## Results

### General subject information

The basic characteristics of the case and the control groups, which contain 495 hypertensive patients and 539 normal blood pressure controls, are shown in Table 1. Sex, age, BMI, TG, LDL, HDL, SBP and DBP were recorded and evaluated. The chi-square test was used for sex, and other quantitative data were compared using the independent-sample T test. There was no significant difference in sex and HDL between the case and the control groups, but age, BMI, TG and LDL suggested that the occurrence of hypertension was affected by complex factors.

**Table 1:** Survey indicators and statistical analysis of the study participants

	Hypertensive	Normal	*p*
Number of people	495	539	
Sex (male/female)	199/296	186/353	0.059
Age (years)	60.01 ± 10.68	53.37 ± 10.36	< 0.001*
BMI (kg/m^2^)	23.88 ± 3.12	22.76 ± 3.12	< 0.001*
Systolic blood pressure (mmHg)	164.86 ± 20.22	117.94 ± 10.38	< 0.001*
Diastolic blood pressure (mmHg)	94.48 ± 12.50	72.79 ± 7.382	< 0.001*
Total triglycerides (mmol/L)	2.24 ± 1.67	1.82 ± 1.81	< 0.001*
Low density lipoprotein (mmol/L)	2.83 ± 0.83	2.56 ± 0.75	< 0.001*
High density lipoprotein (mmol/L)	1.43 ± 0.47	1.42 ± 0.31	0.960

Quantitative data are reported as the mean ± standard deviation and were tested with the independent-sample T test. Qualitative data were analysed by the chi-square test

*BMI* body mass index

*P < 0.05

### SNPs typing and analyses

All samples were typed by multiplex PCR-SNP technology. The call rate of genotyping of all SNPs was more than 90%. The frequency distributions of all 17 SNPs were in Hardy Weinberg Equilibrium (HWE) in both the cases and controls (*P* > 0.003, 0.05/17). The chi-square tests (Table 2) were used to compare the allelic frequency of 17 SNPs on 4 genes in the cases and the controls. There was no significant difference in the *CACNA1C* gene between the cases and the controls. One SNP in CACNA1S, three SNPs in *CACNB2* and one SNP in *CACNA1A* showed significant differences between the case and the control groups. However, after Bonferroni’s correction (*P* < 0.003 (0.05/17)), the results were not significant.

**Table 2:** Comparison of the 17 SNP frequencies between hypertensive and normal populations

Nearby gene	SNP	Chromosome: position	Minor/Major	Hypertensive	Normal	*P*
CACNA1S	rs3767505	1:201065408	C/T	272/658	326/712	0.298
	rs2365293	1:201106117	T/C	335/561	332/678	0.039*
CACNB2	rs10764327	10:18159919	G/T	350/526	427/601	0.484
	rs1757209	10:18183913	G/A	149/775	203/841	0.055
	rs7072759	10:18282286	G/A	404/494	446/570	0.632
	rs17539088	10:18370214	G/A	270/676	343/691	0.026*
	rs16917217	10:18370276	G/A	274/678	343/689	0.032*
	rs61839222	10:18370285	A/C	274/678	343/689	0.032*
CACNA1C	rs2283274	12:2075300	C/G	323/591	366/652	0.779
	rs7304870	12:2164089	A/G	448/422	498/528	0.200
	rs10848627	12:2203242	G/A	296/654	343/703	0.435
	rs10848628	12:2203323	C/T	291/657	341/701	0.331
	rs2239031	12:2227003	T/G	262/680	304/734	0.469
CACNA1A	rs2074880	19:13261817	C/A	353/557	436/580	0.066
	rs10425859	19:13408848	A/G	469/461	474/562	0.038*
	rs76143985	19:13415178	A/G	268/650	265/777	0.062
	rs1422259	19:13428788	C/T	293/627	337/679	0.535

The chi-square test was performed for the 17 loci in the Dai group. *SNP* single nucleotide polymorphism. *P < 0.05, but after Bonferroni’s correction (*P* < 0.003 (0.05/17)), the positive results disappeared

### Logistic regression analysis of association with hypertension

Given that Bonferroni’s correction was too strict, we introduced covariables (adjusted sex, age, BMI, TG, LDL, and HDL) into logistic regression models to adjust the association between polymorphisms and hypertension. Regression analyses (Table 3) were performed under the assumption of three genetic models (dominant, recessive and additive models) for 5 SNPs, which showed significant differences in chi-square tests. The significant associations were found between under the assumption of the dominant model (four SNPs) and the additive model (four SNPs), but no SNPs in the recessive model.

**Table 3:** Relation of SNPs to hypertension as determined by multivariable logistic regression analysis

Nearby gene	SNP	Minor/ Major	Dominant		Recessive		Additive	
			*P*	OR (95% CI)	*P*	OR (95% CI)	*P*	OR (95% CI)
CACNA1S	rs2365293	T/C	0.048*	1.335 (1.002–1.778)	1.102	–	0.093	–
CACNB2	rs17539088	G/A	0.027*	0.732 (0.554–0.966)	0.702	–	0.021*	0.769 (0.616–0.960)
	rs16917217	G/A	0.031*	0.737 (0.558–0.973)	0.719	–	0.025*	0.776 (0.622–0.969)
	rs61839222	A/C	0.031*	0.737 (0.558–0.973)	0.719	–	0.025*	0.776 (0.622–0.969)
CACNA1A	rs10425859	A/G	0.177	–	1.494	–	0.025*	1.269 (1.030–1.563)

Multivariable logistic regression analysis was performed with adjustment for sex, age, BMI, TG, LDL and HDL

*OR* odds ratio, *CI* confidence interval

*P < 0.05

## Discussion

Although the statistically significant SNPs were no longer significant after Bonferroni’s correction, logistic regression still suggested that 5 candidate SNPs consistent with the chi-square tests were associated with hypertension (adjusted sex, age, BMI, TG, LDL, and HDL).

VDCCs mediate the entry of calcium ions into excitable cells. Their function involves the contraction of vascular smooth muscle, the release of neurotransmitters or hormones and the expression of genes. The VDCC is a multi-protein complex composed of a variety of subunits, including α1, β, α2/δ and γ. The activity of VDCCs is mainly directed by the pore-forming α1 subunit, which is the target of calcium antagonist (CCBs) [22, 23],and can be divided into different types, such as α1A, α1B, α1C, α1D, α1E and α1S. In this study, the SNPs selected from the genes encoded subunits alpha 1A (*CACNA1A*), alpha 1C (*CACNA1C*), alpha 1S (*CACNA1S*), and beta (*CACNB2*).

Previous studies have shown that the upregulation of VDCC expression can lead to the enhancement of calcium influx and thereby increase the contractility of VSMCs [24], which partly explains the pathogenesis of hypertension. On the other hand, VDCCs not only regulate the contraction of VSMCs but also affect differentiation, which is also associated with various cardiovascular diseases [25]. Kudryavtseva O et al. [26]. investigated the effect of VDCCs on VSMC differentiation by injecting alpha 1C-siRNA into mice to reduce the expression of VDCCs. Their research showed that the decrease in VDCC expression promoted the change in VSMCs from contractile to non-contractile (the mRNA expression of myosin heavy chain 11 (Myh11), α-actin (Acta2), h-caldesmon was significantly reduced). Therefore, the upregulation or downregulation of VDCC expression can cause a change in VSMC tone.

We found one positive SNP (rs10425859), which suggests an increased risk for the people carried the A_minor_ allele on this SNP. (OR_Additive_ = 1.269 CI95%_Additive_ = 1.030–1.563), in CACNA1A in the Dai population after logistic regression analyses, in CACNA1A in the Dai population after logistic regression analyses. The α1A (*CACNA1A*) subunit is mainly expressed in the nervous tissue of the brain and involves the transmission of synaptic signals in the central nervous system. Research on rat models has shown that abnormal expression of the *CACNA1A* gene can lead to the desensitization of calcitonin gene-related peptide (CGRP) receptors, which are critical in maintaining the systolic response of cerebrovascular smooth muscle [27, 28]. In addition, Hu Z et al. also reported the association between *CACNA1A* polymorphisms (rs8182538) and hypertension in Chinese Han [15].

In Dai people, another positive SNP (rs2365293) was found, which suggests an increased risk for the people carried the T_minor_ allele on this SNP (OR_Dominant_ = 1.335 CI95%_Dominant_ = 1.002–1.778). It was found in *CACNA1S, which encodes* the α_1S_ subunit expressed in skeletal muscle cells. According to the database of Online Mendelian Inheritance in Man (OMIM), the mutations in this gene are associated with hypokalemic periodic paralysis, thyrotoxic periodic paralysis, and malignant hyperpyrexia. A report has shown that this gene is associated with hypertension in genome-wide homozygous imbalance analysis, [29] but the specific mechanism of hypertension has not been reported.

Association analyses in China [14], Lithuanian [17] and Europe [16] have reported the association between *CACNB2* polymorphisms (rs4373814 in Chinese Han, rs12258967 in Lithuanian and rs18748804 in Europe) and hypertension. In our study, we found three other positive SNPs (rs17539088, G_minor_, OR_Dominant_ = 0.732 CI95%_Dominant_ = 0.554–0.966, OR_Additive_ = 0.769 CI95%_Additive_ = 0.616–0.960; rs16917217, G_minor_, OR_Dominant_ = 0.737 CI95%_Dominant_ = 0.558–0.973, OR_Additive_ = 0.776 CI95%_Additive_ = 0.622–0.969; and rs61839222, A_minor_, OR_Dominant_ = 0.737 CI95%_Dominant_ = 0.558–0.973, OR_Additive_ = 0.776 CI95%_Additive_ = 0.622–0.969), which suggest a decreased risk, in CACNB2 after logistic regression analyses. Although the auxiliary subunit coded by *CACNB2* is not the main structure of VDCC activity, it is equally important to maintain the normal structure and function of the whole protein complex. For example, the molecular signals released by the β subunit guide α1 subunit transport to the plasma membrane to construct the correct functional protein complex [30]. In addition, Durairaj Pandian V et al. found that the overexpression of *CACNB2* can upregulate the gene expression of the RAS-MAPK pathway, which is one of the key chain reactions of hypertension [31].

The identification of pathogenic genes of complex diseases such as hypertension has always been difficult. Generally, association analyses are used to screen risk genetic variations of complex diseases. One of the difficulties in association research is that the genetic background of the samples used in the analysis is complex. Due to ethnic and cultural habits, most Dai people are likely to inhabit their villages and rarely intermarry with other ethnic groups in Yunnan Province, leading to the relatively homogenous genetic background of the Dai people. Compared with the Han people who have a complex genetic heterogeneity, the Dai have genetical homogeneity and a smaller pool of susceptibility genes. The disease gene spectrum of hypertension may be narrow in this population. A few disease gene mutations responded for the hypertension in Dai people, which provides an advantage in finding susceptibility variations of hypertension in this special group.

The positive SNPs showed in this study all locate in the intron. These SNPs may be linked to susceptible mutations, which make them show positive results in association analysis. The frequency of susceptible mutations may be low, which makes it difficult to obtain significant positive results by association analysis. However, during evolution, especially in these ethnic groups with a relatively narrow genetic background, the mutations are likely to be linked to Tag SNPs, so that they can be detected in our study. These findings may help us understand the genetic causes of hypertension from a unique perspective.

There are some limitations in the current study. First, as a cross-sectional study, we were not able to determine the causal relationship between VDCCs and hypertension. Second, since all the participants were Dai people, the generalisability of the results may not extend to non-Dai populations. Although the main constraint of this study was the small sample size of the Dai group, the statistical power is enough for the number of analysed SNPs. Further functional studies and association analyses in larger samples and other populations should be conducted to confirm our results.

## Conclusion

In this study, the SNPs identified in the *CACNA1S, CACNB2* and *CACNA1S* genes suggest a different molecular pathogenesis of hypertension in Dai people. Therefore, further studies are needed to verify the functional association between variations in VDCC protein-coding genes and hypertension and susceptibility to hypertension in terms of ethnicity, which may help develop new hypertension diagnoses and treatment methods.

## Supplementary information

**Additional file 1:Supplementary Table S1.** Primer sequences for genotyping the 17 SNPs.

## Data Availability

All data generated or analysed during this study are included in this published article [and its supplementary information files].

## Abbreviations

BMI: Body mass index
CGRP: Calcitonin gene-related peptide
CHB: Chinese Han in Beijing
CI: confidence interval
DBP: Diastolic blood pressure
HDL: High density lipoprotein
HWE: Hardy Weinberg Equilibrium
LDL: Low density lipoprotein
Myh11: Myosin heavy chain 11
NCD-RisC: Non-Communicable Diseases Risk Factor Collaboration
OMIM: Online Mendelian Inheritance in Man
OR: odds ratio
SBP: Systolic blood pressure
SNPs: Single nucleotide polymorphisms
TG: Total triglyceride
VDCCs: Voltage-dependent calcium channels
VSMCs: Vascular smooth muscle cells
