# Supplementary material for: Association between gene polymorphisms of voltage-dependent Ca2+ channels and hypertension in the Dai people of China: a case-control study
Source: BMC Med Genet. 2020 Feb 28;21:44. doi: 10.1186/s12881-020-0982-9 (PMC7049211; doi:10.1186/s12881-020-0982-9)
Supplement: Supplementary file 1 — Additional file 1:Supplementary Table S1. Primer sequences for genotyping the 17 SNPs. [file 12881_2020_982_MOESM1_ESM.docx]

Supplementary Table 1: Primer sequences for genotyping the 17 SNPs

| SNP | Forward | Reverse |
| --- | --- | --- |
| rs3767505 | GTTGCTGTCAGGTTTTCATTTTAG | TCTCTTAAAGCTATTGTCATGCAC |
| rs2365293 | GTATCACCACACATCTGAAGTTG | TGATGGTAAAGATGGCTGTGAG |
| rs10764327 | CTGCGTAGACTGATATGTTTAAGG | AGATGAGTTCTTAGGACACCAAAC |
| rs1757209 | ATAATTGTTTCATCTGCACAGAGG | ATTCTCCTAGATGTGAACCTATGG |
| rs7072759 | GAATGGATGTTTACAGGTCGTAAG | GTGACTGGTCTATCTTTTGTCTATG |
| rs17539088_rs16917217_rs61839222 | CACACTTGTGGCTTCTACTAGACTCC | CAGTGAGCCTACCCAGATAGTGC |
| rs2283274 | TTTGATGATTGCAAGGGATGATAG | AAAGTGTAAAATGTAAGAGGTCCC |
| rs7304870 | TCTCTTTCTTTCTTTAGGTTTGGC | ATAAAATGGTGCAAGGGGATATTC |
| rs10848627_rs10848628 | GCCACAGGAGCGACCAGC | CCCTTCAGTGGGTGAGACAGC |
| rs2239031 | ACTGGCCTAAGGTTTTCAAATATG | GAGAGGAGGGGAATAGCTAAAAAG |
| rs2074880 | ATTTTACAGGCAAGGTCATAAGTC | CTTCACTCAAAAGGATTGAGAGAG |
| s10425859 | GACAGTAGTTGGTCAAAGGAATAC | TTAATAGAGCTCATGACCCATCTC |
| rs76143985 | TGTATTCCCAGGGAGCTTAAAG | ACAGAATGGGCTGAGAAAAATATG |
| rs1422259 | TCCTCCTTTTATGAGTAGGGAAAC | ATCCAAATGTTCAGCTACTTTCTG |

SNP = single nucleotide polymorphism
